# Corals survive severe bleaching event in refuges related to taxa, colony size, and water depth

**DOI:** 10.1038/s41598-024-58980-1

**Published:** 2024-04-18

**Authors:** Erin M. Winslow, Kelly E. Speare, Thomas C. Adam, Deron E. Burkepile, James L. Hench, Hunter S. Lenihan

**Affiliations:** 1 Bren School of Environmental Science and Management, University of California Santa Barbara, Santa Barbara, CA 93106 USA; 2 Department of Ecology, Evolution and Marine Biology, University of California Santa Barbara, Santa Barbara, CA 93106 USA; 3 Marine Science Institute, University of California Santa Barbara, Santa Barbara, CA 93106 USA; 4 Nicholas School of the Environment, Duke University, Beaufort, NC 28516 USA

**Keywords:** Coral bleaching, Thermal stress, Depth, Colony size, *Acropora*, *Pocillopora*, Bleaching refuge, Accumulated heat stress, Temperature fluctuation, Marine biology, Community ecology, Climate-change ecology

## Abstract

Marine heatwaves are increasing in frequency and duration, threatening tropical reef ecosystems through intensified coral bleaching events. We examined a strikingly variable spatial pattern of bleaching in Moorea, French Polynesia following a heatwave that lasted from November 2018 to July 2019. In July 2019, four months after the onset of bleaching, we surveyed > 5000 individual colonies of the two dominant coral genera, *Pocillopora* and *Acropora*, at 10 m and 17 m water depths, at six forereef sites around the island where temperature was measured. We found severe bleaching increased with colony size for both coral genera, but *Acropora* bleached more severely than *Pocillopora* overall*. Acropora* bleached more at 10 m than 17 m, likely due to higher light availability at 10 m compared to 17 m, or greater daily temperature fluctuation at depth. Bleaching in *Pocillopora* corals did not differ with depth but instead varied with the interaction of colony size and Accumulated Heat Stress (AHS), in that larger colonies (> 30 cm) were more sensitive to AHS than mid-size (10–29 cm) or small colonies (5–9 cm). Our findings provide insight into complex interactions among coral taxa, colony size, and water depth that produce high spatial variation in bleaching and related coral mortality.

## Introduction

Human-induced climate change is increasing the frequency and intensity of marine heatwaves that can cause extreme ecological changes in marine communities, especially in tropical latitudes^1^. Of major concern is the impact of sustained, elevated seawater temperature on reef-building corals^2^. Coral bleaching is a stress response of corals to warm water in which the coral host expels the endosymbiotic dinoflagellate microalgae (Symbiodiniaceae)^3^ from its tissues, resulting in the loss of coloration in coral colonies. The mutualism between corals and Symbiodiniaceae is sensitive to small changes in temperature, such that an increase of only 1–2 °C can trigger bleaching^4,5^. In some instances, bleached corals can recover from thermal stress by re-establishing endosymbionts in their tissue^6^, but bleaching often leads to coral mortality. As such, extreme warming events have led to massive pan-tropical coral bleaching and mortality in 1998, 2010, and 2015–2016^4,5,7^. Climate projections forecast that bleaching will become more frequent and more severe as the climate continues to warm, thereby threatening corals and coral reef communities at a global scale^8,9^.

Although bleaching is a major threat to corals worldwide, bleaching does not impact all corals uniformly across a reefscape, even during the most severe marine heatwaves^7,10^. Spatial variation in bleaching can be partially related to local levels of heating, solar irradiance, microrefugia as a function of reef complexity, and differences in physical conditions among microhabitats^11–13^ many of which vary across depth. Corals at shallower depths are exposed to higher solar irradiance where light can act synergistically with temperature to initiate coral bleaching^10,14^. Deeper water can serve as a refuge from bleaching through the attenuation of light and general cooling of water with depth^15,16^. In addition to reprieve from light and warmer surface water, deeper reef habitats experience more intense internal waves^17,18^ that can originate near shelf breaks, travel hundreds of kilometers, and transport cool, nutrient rich water onto the reef^17,19^. Waves and currents can also buffer reefs from the thermal stress associated with high surface temperatures^20^, as water movement facilitates gas exchange that reduces the buildup of oxygen radicals^21–23^.

Different coral genera and associated endosymbionts vary widely in their susceptibility to bleaching, and thus taxonomic composition of the coral community can drive spatial and temporal patterns of bleaching and mortality^24^. Massive and encrusting coral taxa are generally more resistant to bleaching and bleaching-induced mortality in the short term and are therefore considered “winners”, while branching and tabular species are usually more susceptible to heat stress and are often classified as “losers” during bleaching events^10^. Variation in bleaching susceptibility also exists within a given taxa as a function of colony size^25,26^. For example, larger colonies of *Pocillopora* and *Acropora* sometimes exhibit higher bleaching prevalence, severity, and bleaching-induced mortality^25,27,28^ than smaller conspecifics. Additionally, within genera, species may also differ in their susceptibility to thermal stress^15^, and for *Pocillopora*, size-dependent bleaching and mortality may be driven in part by the disproportionate representation of thermally-sensitive cryptic species across the size spectrum^29^.

**Figure 1:**
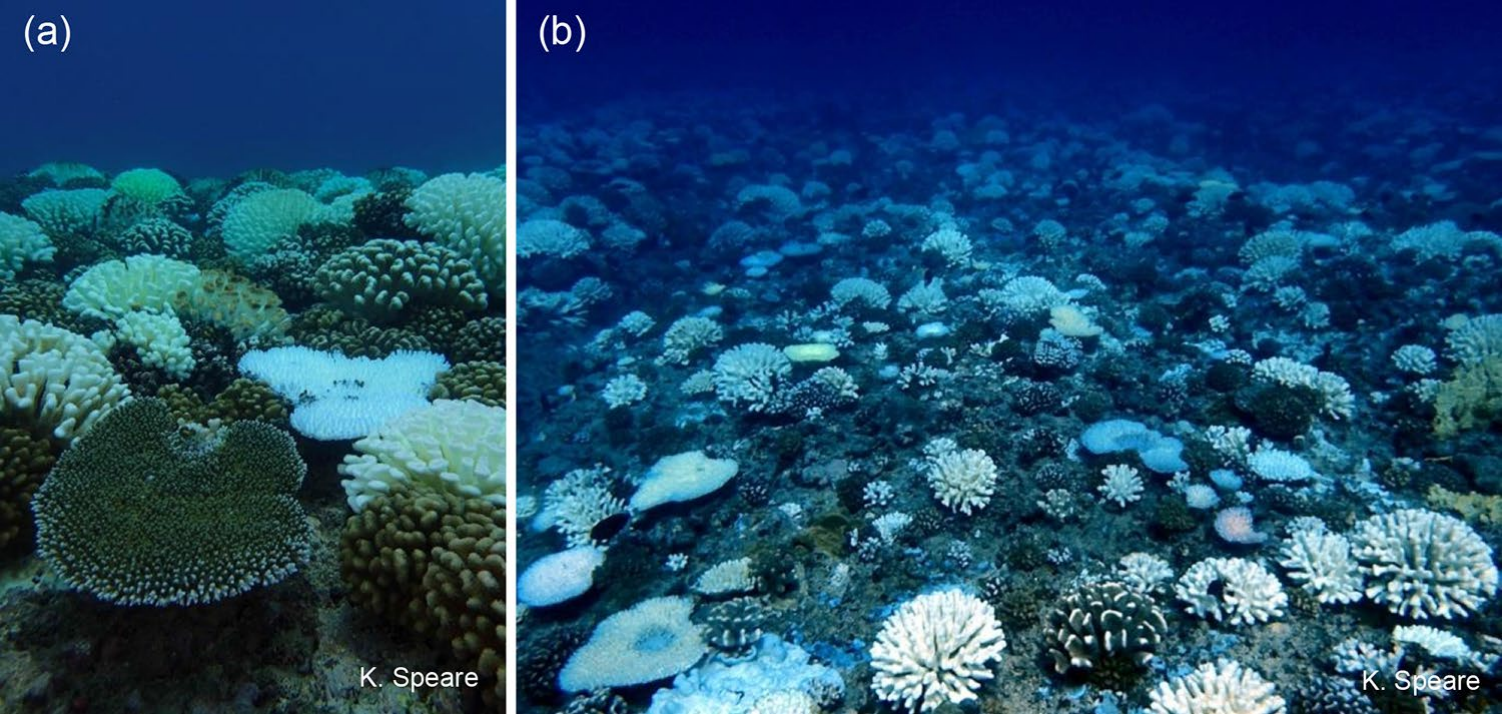
Bleaching across the reefscape in May 2019, two months after bleaching began on (**a**) the north shore (LTER 1) and (**b**) the west shore (LTER 5). Photo credit: Kelly Speare.

Here, we examined how corals on shallow and mid-depth tropical reefs responded to a prolonged marine heatwave that impacted Moorea, French Polynesia from November 2018 to July 2019 to further understand the context that drives variation in the bleaching response in corals (Fig. 1). Using in situ temperature measurements, we quantified how accumulated heat stress (AHS, an integral measure in °C-weeks) and the mean daily temperature fluctuation (MDTF, measured in °C) varied across small spatial scales around the island. We also conducted an extensive survey two months after the peak of the marine heatwave that focused on the two most abundant genera of corals on the outer reef in Moorea, *Pocillopora* and *Acropora*. We assessed how heat stress influenced bleaching patterns in concert with coral taxonomy, colony size, and depth around Moorea, and hypothesized that above a threshold of heat stress, coral bleaching would increase with colony size but decrease with water depth. The results of this study are novel in that they provide evidence that refugia exist for many corals impacted by a marine heatwave and show that patterns of survivorship are shaped by taxonomic identity, water depth, colony size, and the interaction of in situ heat stress with colony size.

## Results

### Seawater temperature

**Figure 2:**
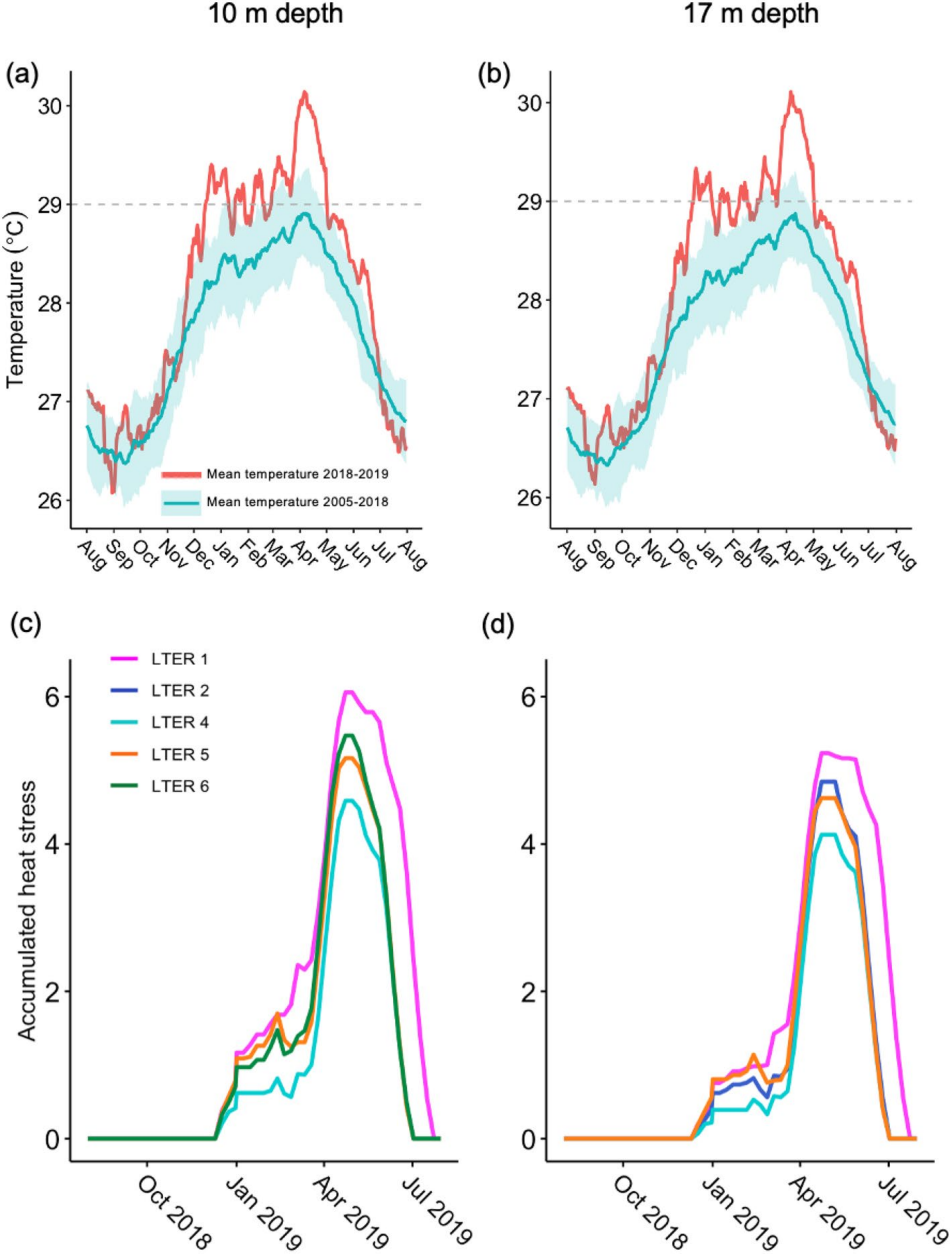
Mean temperature (blue line) ± 95% confidence interval (blue shading) across days from 2005 to 2018, and mean temperature (red line) of the bleaching year from 1 August 2018 to 31 July 2019 at (**a**) 10 m, and (**b**) 17 m. Dotted horizontal line represents the 29 °C threshold temperature at which corals begin accumulating thermal stress. Accumulated heat stress (°C-weeks) at (**c**) 10 m depths and (**d**) 17 m depths from 1 August 2018 to 31 July 2019 at the sites used in our study.

During the prolonged marine heatwave from November 2018 to July 2019, seawater temperatures (measured in situ) were consistently above the previous long-term mean ocean temperature by at least one standard deviation at both 10 and 17 m water depths (Fig. 2). Seawater temperatures were above 29.0 °C from 14 December 2018 until 1 May 2019 at both 10 and 17 m, which is the threshold above which corals in Moorea begin experiencing thermal stress^30^. Bleaching was first reported in Moorea in March 2019 after 88 days of accumulated heat stress, defined in the paragraph below. Mean daily seawater temperature was marginally higher at 10 m (29.31 ± 0.06 °C, mean ± SD) than 17 m (29.22 ± 0.05 °C) pooled across forereef sampling sites (see Supplementary Fig. S1 for sites; t_6_ = 2.03, *p* = 0.08, 95% CI [− 0.017, 0.184]; Fig. 2). The maximum mean daily seawater temperature at 10 and 17 m sites was 30.30 °C and 30.27 °C respectively. It is important to note that four of the twelve temperature loggers failed to collect continuous temperature data throughout the duration of the bleaching year and we omitted those site x depth combinations from our analysis (see Supplementary Fig. S1). We acknowledge the instrumentation failure potentially limits our ability to detect nuanced patterns in temperature dynamics. See “Materials and Methods” for specific details.

Accumulated heat stress (AHS) is a measure of the magnitude and duration of warming that corals experience^30^, and is defined as the number of weeks over a threshold temperature (29 °C in Moorea) in a 12-week running sum with units of °C-weeks (see “Materials and Methods”). Prior work in Moorea found that maximum AHS above 4.0 °C-weeks resulted in bleaching in 1991, 1994, 2002, 2003, and 2007^30^. During the bleaching year, 1 August 2018 to 31 July 2019, maximum AHS across sites was not significantly higher at 10 m depth (5.32 ± 0.61 °C-weeks, mean ± SD) than 17 m (4.71 ± 0.46 °C-weeks) (t_6_ = 1.60, *p* = 0.16, 95% CI [-0.327, 1.553]). However, the maximum AHS observed at any 10 m site was 6.06 °C-weeks as compared with a maximum value of 5.24 °C-weeks at 17 m. The northwestern most site (LTER 1) experienced the highest level of AHS at both 10 and 17 m and the southeastern site (LTER 4) was the coolest at both depths (Table 1; Fig. 2). For each site where continuous measurements were collected at both 10 and 17 m, the deeper site experienced lower AHS (Fig. 2).

**Table 1:** Maximum accumulated heat stress (AHS, measured in °C-weeks) and mean daily temperature fluctuation (MDTF) values for each site x depth used in the analysis. AHS values were calculated for site x depth combinations with continuous temperature data during the bleaching year, 1 August 2018 to 31 July 2019. MDTF values were calculated for the month leading up to the first signs of bleaching (15 February to 15 March 2019), averaged across days.

Site	Depth	Maximum accumulated heat stress (°C-weeks)	Mean daily temperature fluctuation (°C)
LTER 1	Mid (10 m)	6.06	0.45
	Deep (17 m)	5.24	0.59
LTER 2	Deep (17 m)	4.85	0.67
LTER 4	Mid (10 m)	4.59	0.41
	Deep (17 m)	4.13	0.56
LTER 5	Mid (10 m)	5.17	0.29
	Deep (17 m)	4.62	0.39
LTER 6	Mid (10 m)	5.47	0.39

Exposure to short-term high frequency temperature fluctuations prior to a warming event can bolster thermal tolerance in scleractinian corals and enable colonies to escape bleaching^31–33^. Mean daily temperature fluctuation (MDTF, °C) in the 30 days prior to the onset of bleaching can have a strong mitigating effect on bleaching^32^. Bleaching was first observed in mid-March 2019, and we selected 15 March as the start of the bleaching event for our analyses. We found that, on average, our four 17 m sites experienced a higher MDTF (0.55 ± 0.12 °C, mean ± SD) than the 10 m sites (0.38 °C ± 0.07 °C); (t_6_ = − 2.47, *p* < 0.05, 95% CI [− 0.335, − 0.001]; Supplementary Fig S2) from 15 February to 15 March. The northern sites (LTER 1 and 2) had the highest MDTF values and the western sites (LTER 5 and 6) had the lowest values (Table 1). For each site where continuous measurements were collected at both 10 and 17 m, the deeper site experienced a higher MDTF (Table 1).

### Bleaching patterns

**Figure 3:**
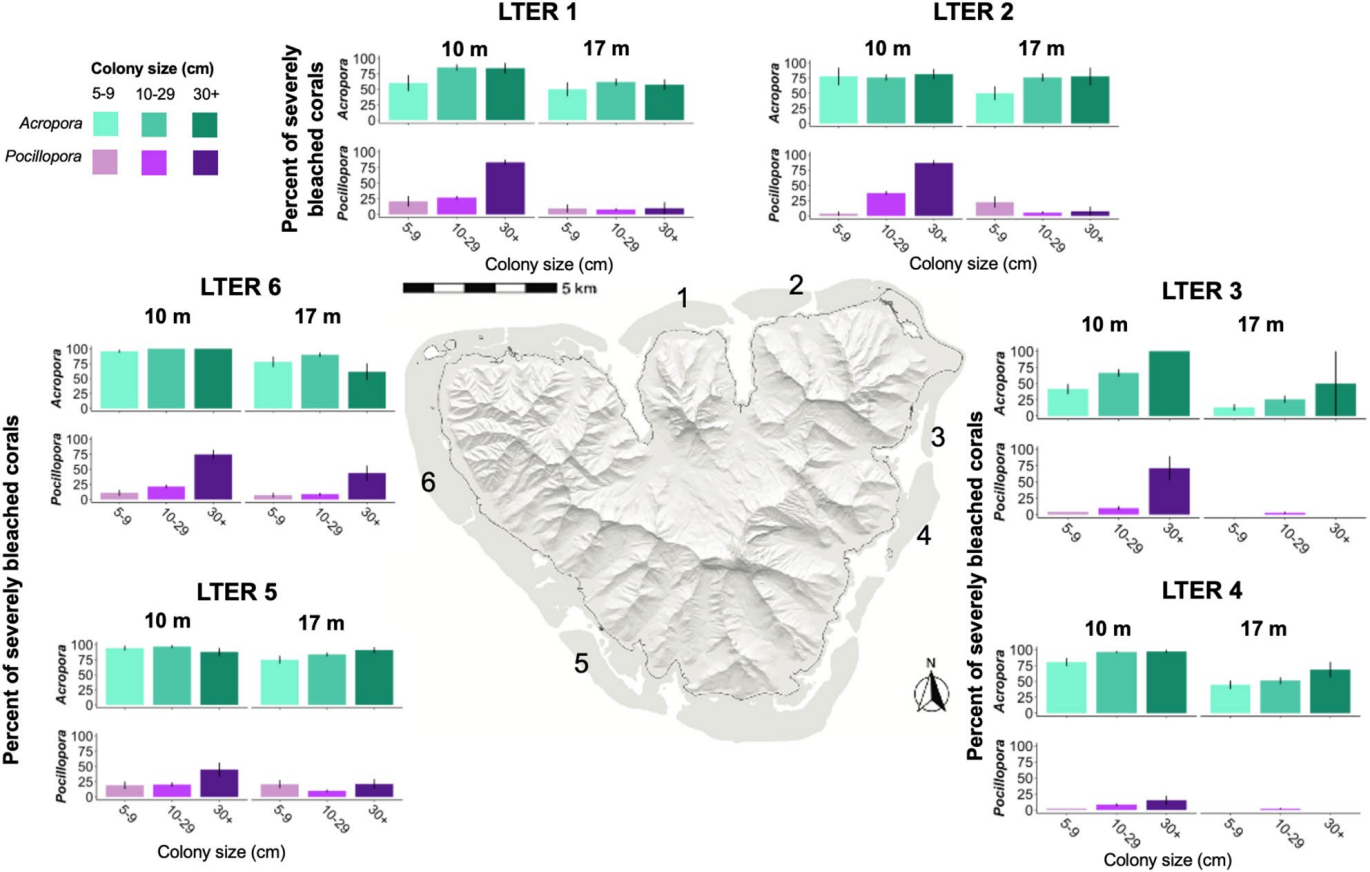
Percent of individuals severely bleached (≥ 75% of colony bleached and/or dead) in July 2019 across depth at the six permanent LTER sites around the island for both *Acropora* and *Pocillopora* corals. Green hues represent *Acropora* corals in 5–9 cm, 10–29 cm, and large ≥ 30 cm size classes. Purple hues represent *Pocillopora* in 5–9 cm, 10–29 cm, and large ≥ 30 cm size classes. Error bars represent standard error. Land is displayed as a digital elevation model. Shallow lagoon habitat surrounding the island is shown in gray. Surveys were conducted on the steeply sloping forereef immediately offshore of the shallow lagoons. The map was created in R version 4.0.2^34^ using the ggplot2^35^ and raster^36^ packages.

In our survey of 5,101 corals around the island and across depth, we found that overall, more *Acropora* corals (n = 1874) bleached than *Pocillopora* corals (n = 3227; Fig. 3). To understand which genera bleached more severely, we defined severely bleached corals as colonies that were ≥ 75% bleached and/or dead at the time of our surveys in July 2019. Overall, 79.9% ± 1.21 (mean ± SE) of *Acropor*a colonies bleached severely compared to 18.1% ± 0.90 (mean ± SE) of *Pocillopora* colonies. There was a distinct pattern of size-dependent bleaching at most sites around the island for *Pocillopora* corals, especially at 10 m, where larger corals (≥ 30 cm in diameter; 65.6% ± 3.80 of colonies, mean ± SE) bleached more severely than mid-size (10–29 cm; 19.8% ± 1.52 of colonies, mean ± SE) and smaller corals (5–9 cm; 12.8% ± 2.74 of colonies, mean ± SE; Fig. 3). Fewer *Pocillopora* corals overall bleached at 17 m where the pattern of size-dependent mortality was weaker. In general, most *Acropora* corals bleached severely on the north (67.8% ± 2.80 of colonies, mean ± SE) and southwest (91.3% ± 1.29 of colonies, mean ± SE) sides of the island (Fig. 3). On the east side of the island (LTER 3 and 4) where AHS was lower, observed patterns in *Acropora* bleaching were more size-dependent where large corals (≥ 30 cm in diameter; 88.9% ± 4.32 of colonies, mean ± SE) bleached more severely compared to mid-size (10–29 cm; 76.7% ± 3.02, mean ± SE) and small (5–9 cm; 58.9% ± 5.22, mean ± SE) colonies (Fig. 3). *Pocillopora* corals also bleached less severely on the east side of the island (5.47% ± 1.14 of colonies, mean ± SE) with very little bleaching of any size classes observed at 17 m (Fig. 3). While more *Acropora* colonies bleached than *Pocillopora* colonies at all sites, *Acropora* appear to have experienced a depth refuge at 17 m where 66.4% ± 0.02 (mean ± SE) of colonies bleached severely compared with 10 m where 93.3% ± 0.01 (mean ± SE) of colonies bleached severely (Fig. 3).

### Drivers of severe bleaching in* Acropora* corals

**Figure 4:**
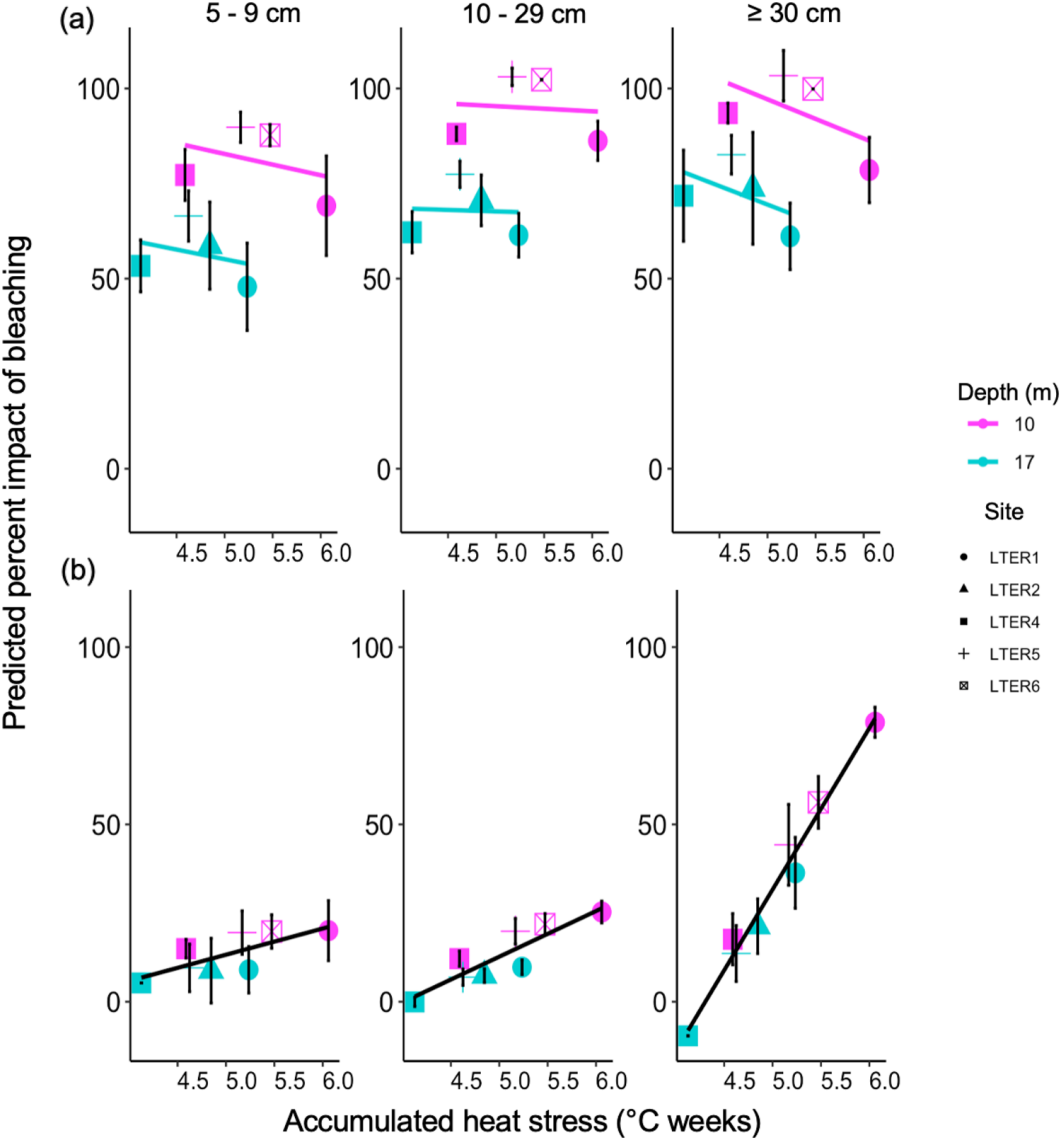
Linear mixed model outputs of the predicted mean percent of severely bleached corals at each site and depth for colonies 5–9 cm, 10–29, and ≥ 30 in diameter for (**a**) *Acropora* corals, and (**b**) *Pocillopora* corals across measured accumulated heat stress (AHS, measured in °C-weeks) values. Pink represents model outputs for 10 m depth, teal represents model outputs for 17 m depth, and shapes represent the different sites. Error bars represent standard error.

Severe bleaching in *Acropora* corals was significantly related to depth (Depth effect: χ^2^(1) = 11.24, *p* = 0.014; Supplementary Table S1) and size (Size effect: χ^2^(2) = 6.32, *p* = 0.011; Supplementary Table S1), but not AHS (AHS effect: χ^2^(1) = 0.41, *p* = 0.558; Supplementary Table S1), nor the interaction between size and AHS (Size x AHS effect: χ^2^(2) = 0.65, *p* = 0.535; Supplementary Table S1). *Acropora* corals at 10 m, (92.5% ± 6.43 of colonies), bleached more severely than those at 17 m (65.2% ± 6.43 of colonies; *p* = 0.014; Fig. 4; Supplementary Table S1). Small *Acropora* corals (5–9 cm; 69.9% ± 5.41 of colonies) bleached less severely than both mid-size (10–29 cm; 82.5% ± 5.41) and large colonies (≥ 30; 84.2% ± 5.41; pairwise comparison of marginal means, *p* = 0.031 and *p* = 0.015 respectively; Fig. 4; Supplementary Table S1), regardless of depth. The effect of AHS was not significant for *Acropora* corals, but this observation was probably driven by *Acropora* corals bleaching severely at even the lowest observed levels of AHS.

### Drivers of severe bleaching in* Pocillopora* corals

There was no significant effect of water depth on severe bleaching of *Pocillopora* corals (Depth effect: χ^2^(1) = 1.61, *p* = 0.238; Supplementary Table S2) or AHS on severe bleaching (AHS effect: χ2(1) = 9.60, *p* = 0.10; Supplementary Table S2). Patterns of bleaching in *Pocillopora* corals differed from *Acropora* corals in that there was a significant interaction between size and AHS driving bleaching in *Pocillopora* (Size × AHS effect: χ^2^(2) = 8.60, *p* = 0.004; Supplementary Table S2). This interaction was significant because large (≥ 30 cm) *Pocillopora* colonies were more sensitive to AHS than mid-size (10–29 cm) and small-(5–9 cm) colonies, where 32.1% ± 4.25 of large colonies bleached severely compared with 13.4% ± 4.25 and 13.0% ± 4.25 of small and medium colonies for the same level of AHS (pairwise comparison of marginal means, *p* = 0.010 and *p* = 0.011 respectively; Fig. 4; Supplementary Table S2).

## Discussion

As marine heatwaves increase globally in frequency and duration, the severity and prevalence of coral bleaching is projected to increase^4,8^. However, even in extreme warming events, bleaching can be heterogeneous across taxa and spatially across the reefscape^7^. The idea that certain coral taxa or functional groups are winners or losers in a bleaching event is well-documented^10^. Corals in the genus *Acropora* are known to be highly susceptible to thermal stress, often bleaching at lower levels of heat stress than other genera^37,38^. In 2019 and in past bleaching events in Moorea^25,30^, *Pocillopora* and *Acropora*, the two dominant branching coral taxa in Moorea, bleached extensively around the island. However, there were instances around the island where *Acropora* and *Pocillopora* did not bleach. Our work highlights where each coral taxa avoided bleaching and disentangles some of the factors ameliorating bleaching. Specifically, we found that two coral genera exhibited differential patterns of bleaching amongst size classes and across depth. Understanding the nuanced differences in bleaching across space, colony size, thermal stress, and water depth for different taxa is key to predicting the ultimate impacts of marine heatwaves.

Our work is one of a growing number of studies to focus on smaller-scale variation in temperature measured in situ to explain variation in bleaching across space^39,40^. Using in situ water temperature measurements can be advantageous in identifying variability across small spatial scales because water temperature often varies with depth. We found the maximum AHS values between 10 and 17 m depths at any given site differed by a minimum of 0.46 and maximum of 0.82 °C-weeks above 29.0 °C, which could have major biological implications. Historically, remotely sensed sea surface temperature (SST) has been used to quantify thermal stress experienced by corals and has served as a useful and accessible tool for predicting bleaching events across regional and global spatial scales^41^. For example, Donovan et al^42^. demonstrated that high levels of bleaching from 2006 to 2016 matched high levels of heat stress across 223 sites across the globe. Recent work in the Caribbean related levels of localized coral bleaching to thermal stress across 5 × 5 km^2^ pixels of sea surface temperature^43^. Thus, remotely sensed observations and in situ temperature measurements are both useful tools for detecting thermal stress in corals and each serve distinct purposes in understanding temperature dynamics.

A decline in bleaching severity with water depth is a commonly observed phenomenon following marine heatwaves and is generally attributed to the attenuation of light^44–47^ and temperature^1^ with depth. In our study, *Acropora* bleached less at the deeper sites, a pattern also observed during two previous bleaching events in 1994^48^ and 2002^49^. However, in our study, this pattern could not be explained by a depth-related decrease in temperature stress, at least in the form of AHS. In fact, we did not find bleaching in *Acropora* to be related to AHS, probably because of the high level of heat stress observed across all sites and depths. We propose two alternative hypotheses for how depth could have ameliorated bleaching in *Acropora* corals. First, as noted previously, differences in solar irradiance may be an important driver of differences in bleaching susceptibility across depths. High levels of solar irradiance can result in the production of reactive oxygen species which can damage symbionts and the coral host^50^. Excess solar energy also contributes to higher rates of photoinactivation, which inhibits symbiont photosynthesis^50^, and once symbionts are no longer nutritionally contributing to the symbiosis, the coral host expels them from its tissues^46^. Anomalously warm water can also interact with solar irradiance to exacerbate bleaching, as photosynthesis is a temperature dependent process where photosynthetic rates can drop drastically with warmer temperature^1^, making shallow water corals vulnerable to bleaching. While we did not measure solar irradiance in our study, shading can protect corals against harmful solar radiation^15,51^. Coelho et al.^51^ found that shaded *Acropora* colonies bleached less than those exposed to direct sunlight after only 2-degree heating weeks (DHW). Shaded *Pocillopora* corals only experienced bleaching reprieve after 7 DWH^51^, thus indicating that reductions in light appear to have a greater impact on the bleaching response in *Acropora* compared with *Pocillopora* corals. In addition to differences in light, the greater MDTF at 17 m may have provided *Acropora* colonies an effective depth refuge through temperature acclimation. Relatively large temperature fluctuations at 17 m, probably driven by internal waves^17,19^, in the 30 days leading up to the bleaching event may have acclimated *Acropora* colonies and their endosymbionts to mild temperature stress, as has been documented in other bleaching events^32,52,53^.

Biotic refuges can also provide shelter from disturbance for individuals with traits that enable them to evade the effects of a given perturbation^54^. Corals experience biotic refuge from bleaching through taxonomy, size, morphology, and genetic lineage, amongst other factors^10,29,55^. In our study, *Acropora* and *Pocillopora* showed marked differences in bleaching severity. *Acropora* is known to be a highly susceptible genus to bleaching and has been observed to bleach more than *Pocillopora* historically in Moorea^30^ and globally^56^, although there are exceptions. There is continuous extension between polyps in *Acropora* colonies^57^, which may, in part, contribute to their vulnerability to bleaching. *Acropora* corals are the most physiologically integrated genus in Scleractinia^57^, such that damage to one polyp is not contained and can spread across the colony. Our previous work shows both *Pocillopora* and *Acropora* corals exhibit size-dependent bleaching at 10 m in Moorea^25^, specifically that larger corals bleached disproportionately more than smaller corals. Here, we expand upon this result to show that size was a more important predictor of bleaching than depth for *Pocillopora*. Pooled across all sites and depths, 52% of large *Pocillopora* corals (≥ 30 cm) were severely bleached or dead compared with 13% of small (5–9 cm) individuals and 14% of mid-size (10–29 cm) individuals. Large *Pocillopora* colonies were more sensitive to AHS and bleached disproportionately more than small and mid-size colonies for the same level of AHS. Similarly, our statistical model highlighted that small (5–9 cm) *Acropora* colonies bleached substantially less than mid-size (10–29 cm) and large individuals (≥ 30 cm) across site and depth.

Several mechanisms may contribute to the strong size-dependency of bleaching experienced by corals. Larger corals have a low surface area to volume ratio relative to smaller colonies and are less successful in exchanging compounds with the surrounding seawater^58^, a potential explanation as to why larger corals of both genera bleached more severely than small corals across depth around the island. It is also possible that large *Pocillopora* corals were disproportionately represented by a faster growing, more thermally sensitive cryptic species in Moorea^29^, which may have contributed to the size-dependent bleaching pattern we observed. However, size-dependent bleaching has been observed for other coral genera. For example, following a bleaching event in 2000, large *Oculina* colonies experienced the most bleaching related mortality compared to smaller individuals in the Mediterranean^27^. Similarly, in the Maldives, three species of *Acropora* exhibited size dependent bleaching where larger colonies bleached more than smaller colonies^59^.

As extreme thermal anomalies increase in time and space in our changing climate, sensitive coral species on shallow reefs will continue to suffer extreme rates of mortality. The loss of live corals at shallow and mid-depths from bleaching will likely decrease the diversity and abundance of reef fish^60^ and other invertebrates^61^ that rely on corals for habitat and food, resulting in unpredictable outcomes for tropical marine ecosystems. Existing literature suggests that deep, mesophotic reefs (≥ 30 m) have the potential to sustain coral populations through partial escape from the impacts of warming^62–64^. Our work suggests a shallower (17 m) bleaching reprieve may exist for *Acropora* corals, and although depth did not explain bleaching patterns in *Pocillopora*, larger colonies may find reprieve at depth through the attenuation of temperature. The loss of the largest *Pocillopora* and *Acropora* will likely have unforeseen impacts on reef ecosystems by reshaping the size-structure of populations^25^. This study highlights that reefs do not necessarily experience the wholesale loss of corals even during extreme heatwaves, and that corals have the capacity to escape bleaching and persist into the future. Our work contributes to understanding the intertwined mechanisms whereby corals experience refuge from thermal events, and by disentangling the complex biological and physical factors that drive individual corals to bleach, we are better able to understand the dynamics of bleaching events.

## Materials and methods

### Study site

Moorea, French Polynesia is a high volcanic island (17°30°S, 149°50°W) within the Society Island Archipelago that is characterized by shallow fringing reefs (2–4 m water depth), relatively deep bays (10–35 m), and outer reefs with steeply descending slopes. Our study was conducted on the outer reefs of Moorea which, at the time of the bleaching event, were recovering from a 2007–2009 crown-of-thorn seastar (COTS), *Acanthaster plancii*, outbreak and a cyclone in 2010^37,65,66^. The coral community on the outer reef has historically been a resilient system as it has recovered relatively quickly from other multiple disturbances over the past several decades^67,68^. Bleaching events in Moorea have also been documented in 1991, 1994, 2002, 2008, 2016, and 2017^48,49,69^, several of which were characterized by high levels of spatial variation in coral mortality around the island and across depths. Prior to the 2019 thermal stress event, live coral cover ranged from 13 to 80% (mean 47%) at 10 m water depth and from 12 to 36% (mean 26%) at 17 m depth^70^.

### Ocean temperature

Ocean temperature was recorded continuously around the island on the outer reef as part of the Moorea Coral Reef Long Term Ecological Research (MCR LTER) project from 2005 to 2019^71^. We used measurements recorded at two water depths, 10 and 20 m, at six permanent sites that are monitored by the MCR LTER (henceforth sites are referred to as “LTER 1–6”) (Supplementary Fig. S1). Each site-depth combination contained a bottom-mounted thermistor (SeaBird Electronics SBE 39 or SBE56) that recorded water temperature every 20 min during the 14-year time series. Four of the twelve thermistors failed to collect continuous temperature data throughout the duration of the bleaching year analyzed (1 August 2018 to 31 July 2019), therefore we omitted them from our temperature analysis. Due to this instrument failure, we excluded both 10 and 17 m at LTER 3; 17 m at LTER 6; and 10 m at LTER 2 (see Supplementary Fig. S1 for the specific depth × site locations utilized). To determine whether seawater temperature was warmer at 10 m than 17 m during the warming event (25 November 2018–19 May 2019), we calculated the daily mean seawater temperature at each site-depth combination and performed a Welch two-sided t-test across the 10 m and 17 m sites with continuous temperature data throughout the bleaching year.

#### Accumulated heat stress

To quantify the effect of temperature on bleaching severity in Moorea, we used seawater temperature data to calculate accumulated heat stress (AHS) in °C-weeks, the number of weeks in a 12-week running sum above a maximum monthly mean temperature (°C). The maximum monthly mean (MMM) temperature in Moorea is 29 °C, which is the temperature threshold at which corals begin experiencing thermal stress^30^. The MMM was calculated by Pratchett et al. 2013 using NOAA sea surface temperature and we acknowledge that corals at different depths and locations may have different MMMs^30^. We calculated the AHS as a 12-week running sum of mean weekly temperatures exceeding the MMM (29.0 °C) by at least 0.1 °C at each site × depth combination^30,72^. To test whether maximum AHS was higher at 10 m compared with 17 m during the bleaching year, 1 August 2018 to 31 July 2019, we extracted the maximum AHS value at each site × depth and performed a Welch two-sided t test across 10 m and 17 m sites. We also utilized maximum AHS values in our linear mixed model analyses.

#### Mean daily temperature fluctuation

The mean daily temperature fluctuation (MDTF, measured in °C) is the mean daily range in temperature over a given period of time. The MDTF in the 30 days leading up to the start of bleaching can have a strong mitigating effect on bleaching^32^. Bleaching was first observed in Moorea in mid-March and although we recognize the exact date of bleaching onset is unknown, we used 15 March as the beginning of the bleaching event in our analyses. Temperature fluctuation was calculated for each day from 15 February 2019 to 15 March 2019, the month leading up to the start of the bleaching event, at each site × depth combination. We then took the mean at each site × depth and used a Welch two-sided t test to test whether MDTF was significantly different between 10 and 17 m depths. Due to co-linearity with AHS and depth, we were unable to include MDTF in our linear mixed models (see Supplementary Fig. S3).

### Coral bleaching surveys

To assess whether bleaching severity differed around the island and with water depth, two SCUBA divers conducted benthic surveys along 10 and 17 m isobaths at each of the permanent MCR LTER outer reef sites (LTER 1–6) from 9 to 15 July 2019, approximately two months following the peak in thermal stress (Supplementary Fig. S1). The bleaching and mortality surveys were executed along two belt transects (each 50-m long × 1-m wide) at each depth × site to assess how the marine heatwave impacted coral populations across depth and space. We recorded data on colony-level bleaching and mortality for a total of 5101 colonies of the two dominant coral taxa, *Pocillopora* (n = 3227) and *Acropora* (n = 1874) across 16 transects (two at each depth × site), recording the percent of each individual colony that was healthy, bleached, or recently dead. We defined recently dead as any portion of an individual coral colonized with filamentous turf algae but not yet colonized by macroalgae (Supplementary Fig. S4). The maximum diameter of each colony was estimated visually and assigned a categorical size bin to represent corals of 5–9 cm, 10–29 cm, or ≥ 30 cm in diameter.

We applied a slightly different survey methodology for each genus to account for the greater abundance of *Pocillopora* corals than *Acropora* corals on the outer reef. Each genus was surveyed along a 50 m transect at each site × depth, but only *Pocillopora* colonies that intersected the transect were recorded. Because *Acropora* colonies were less abundant than *Pocillopora*, divers recorded every *Acropora* colony within a 1-m swath along the transect. *Pocillopora* corals cannot reliably be classified to species based on size, color, or morphology^73^ and were therefore identified to genus in our surveys. We acknowledge that several cryptic species exist within the *Pocillopora* genus that have different bleaching responses^29^. Some of the most common *Acropora* corals on the outer reef of Moorea prior to the bleaching event were *Acropora lutkeni*, *A. globiceps*, *A. retusa*, and *A. hyacinthus*^74^*.* We identified *Acropora* colonies to genus as we could not distinguish between most species when bleached or dead. We also acknowledge that species-level differences in bleaching and mortality are possible, however, it is common to pool data by genus for landscape- and regional-scale ecological studies, including those that investigate size-specific or depth-specific impacts of disturbances on corals^75^.

### Statistical analyses

For each site × depth × size class (24 total) combination we calculated the percent of corals that were severely bleached for each genus. Severely bleached corals were individuals that were at least 75% bleached and/or dead at the time of our July surveys. We selected the 75% threshold to consider only individuals that suffered the most extreme impacts of the heatwave. We used a linear mixed model (LMM) for each taxon to evaluate how the percent of severely bleached *Pocillopora* and *Acropora* corals was related to accumulated heat stress (AHS), water depth (10 and 17 m), colony size (an ordinal variable in which 5–9 cm < 11–29 cm <  ≥ 30 cm), and the interaction between AHS and colony size. Water depth, AHS, and colony size were fixed effects in our LMM models, and site was included as a random effect. We excluded sites from our analysis that did not have continuous temperature data from the bleaching year, therefore, our analysis included data from four sites at 10 m and four sites at 17 m sites (see Supplementary Fig. S1 for specific site × depth combinations used). Each taxa-specific model was fit by Restricted Maximum Likelihood (REML) and we ran Type II Wald F tests with Kenward-Roger degrees of freedom approximations on our model to quantify the relative importance each interaction and main effect had in influencing severe bleaching in corals. Post-hoc pairwise tests were performed with the emmeans package using a Tukey adjustment for approximating p values^76^. All data visualization was performed in ggplot2^35^ and model plots through the sjPlot package^77^. In addition to our data analysis at the site level using LMMs, we also analyzed our data on individual corals using Generalized Linear Mixed Models (GLMMs) with a binomial distribution. In this analysis the response variable indicated whether an individual coral colony was severely bleached (1) or not severely bleached (0). We modeled the data separately for each taxa and models included same fixed and random effects as the LMMs. The results of the GLMMs were qualitatively the same as the results of the LMMs and therefore we present the results of the LMMs here.

### Supplementary Information

Supplementary Information.

## Data Availability

The coral bleaching dataset analyzed during the current study is available in this repository and this repository for seawater temperature.
