# Supplementary material for: Corals survive severe bleaching event in refuges related to taxa, colony size, and water depth
Source: Sci Rep. 2024 Apr 18;14:9006. doi: 10.1038/s41598-024-58980-1 (PMC11026537; doi:10.1038/s41598-024-58980-1)
Supplement: Supplementary file 1 — Supplementary Information. [file 41598_2024_58980_MOESM1_ESM.pdf]

Supplementary Materials

Corals survive severe bleaching event in refuges related to taxa, colony size, and water depth

Erin M. Winslow[1†], Kelly E. Speare[2], Thomas C. Adam[3], Deron E. Burkepile[2,3], James L. Hench[4], and Hunter S. Lenihan[1,3]

[1]Bren School of Environmental Science and Management, University of California Santa Barbara, Santa Barbara, CA 93106, USA

[2]Department of Ecology, Evolution and Marine Biology, University of California Santa Barbara, Santa Barbara, CA 93106, USA

[3]Marine Science Institute, University of California Santa Barbara, Santa Barbara, CA 93106, USA

[4]Nicholas School of the Environment, Duke University, Beaufort, NC 28516, USA

† Corresponding author email: erinmwinslow@gmail.com

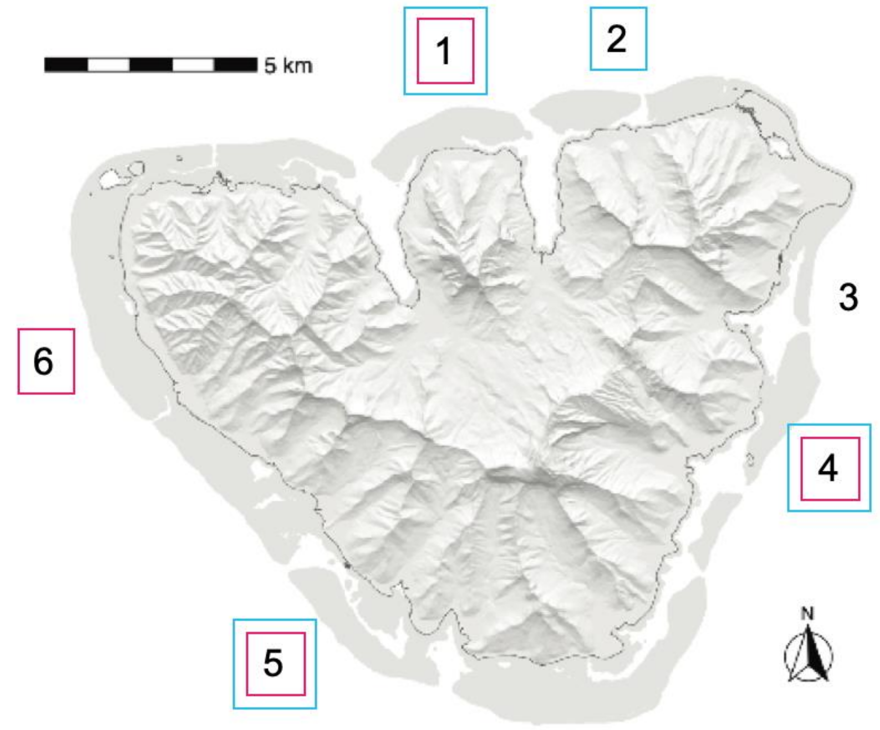

Supplementary Figure S1: Locations of the six permanent LTER sites around Moorea. Divers completed bleaching surveys at 10 and 17 m depths at all six sites, but we only utilized sites with continuous temperature data from 1 August 2018 to 31 July 2019. Sites with continuous temperature data at 10 m are indicated with a pink box, and sites with continuous temperature data at 17 m are indicated with a blue box. Thermistors at both 10 m and 17m failed at LTER 3 resulting in no temperature data from this site. Land is displayed as a digital elevation model. Shallow lagoon habitat surrounding the island is shown in gray. Surveys were conducted on the steeply sloping forereef immediately offshore of the shallow lagoons. The map was created in R version 4.0.2[1] using the ggplot2[2] and raster packages[3].

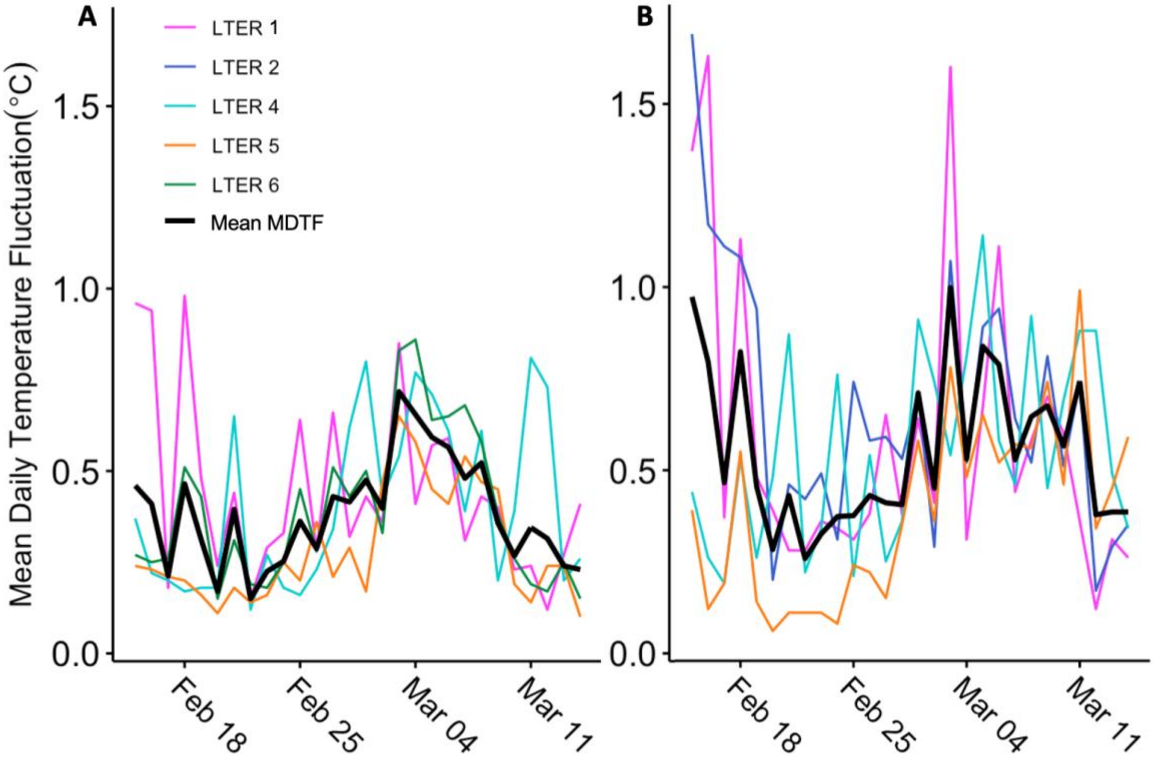

Supplementary Figure S2. Mean daily temperature fluctuation (MDTF) in the 30 days (February 15 – March 15, 2019) leading up to the first signs of the bleaching event at **A)** 10 m and **B)** 17 m at each site. Solid black line represents the mean daily temperature fluctuation (MDTF) across sites at each depth during this time-period.

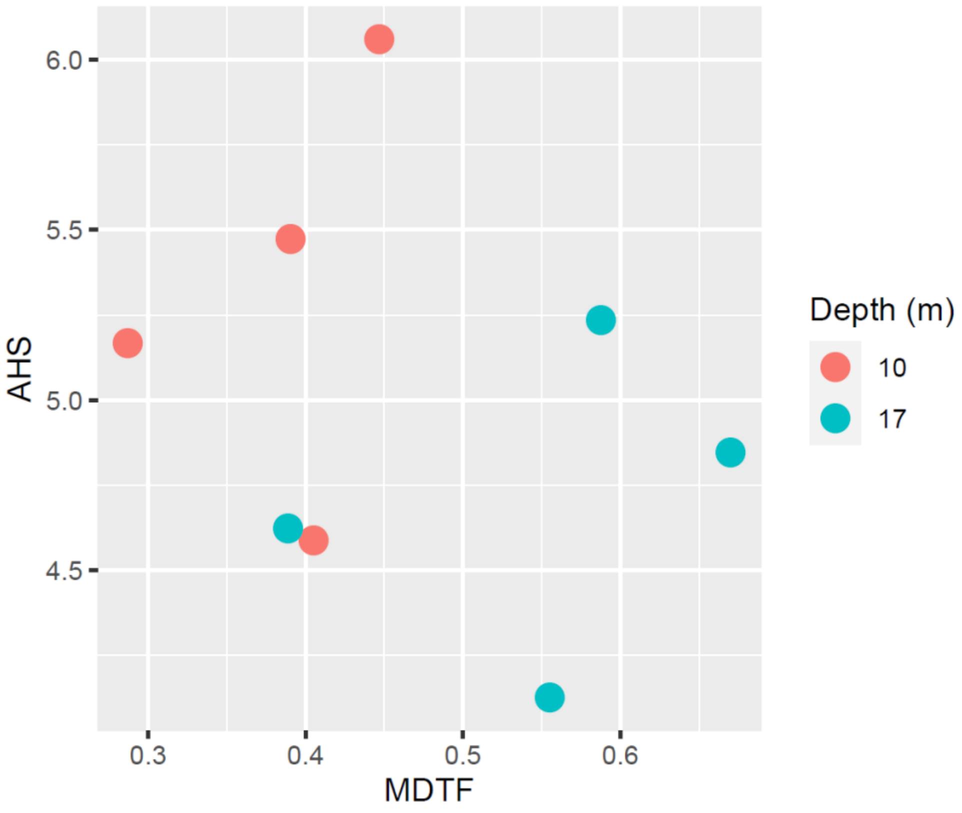

Supplementary Figure S3: Accumulated Heat Stress (AHS) plotted against Mean Daily Temperature Fluctuation (MDTF) at four sites at 10 m depth and four sites at 17 m depth on the forereef of Moorea (values and site names are presented in Table 1 in the main manuscript.

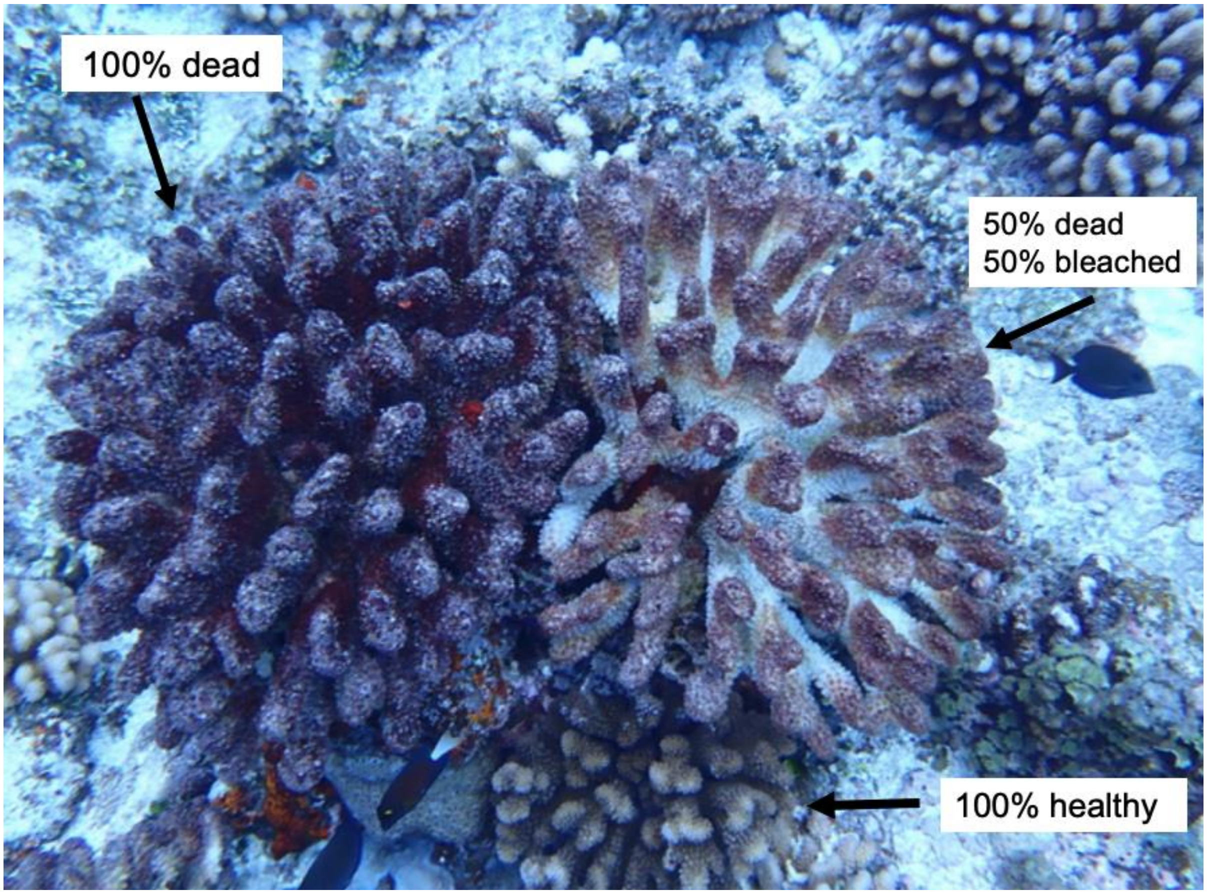

Supplementary Figure S4: Examples of *Pocillopora* impacted by the bleaching event. The colony on the left was considered 100% dead from recent bleaching and the colony on the right was 50% dead and 50% bleached. Thus, both colonies were considered severely bleached (75% bleached and/or recently dead). The bottom coral was 100% healthy and not considered severely bleached. Image modified from Speare et al. 2022.

Supplementary Table S1: Severe bleaching linear mixed model (LMM) results for *Acropora* corals. **A)** random effects, **B)** Wald Chi-square test of LMM, **C)** Pairwise differences in predicted severe bleaching means for *Acropora* corals of different colony sizes, and **D)** pairwise differences of predicted severe bleaching means in *Acropora* corals for different depths. Pairwise comparisons were calculated using a Tukey HSD (honest significant difference) test. SE: standard error; DF: degrees of freedom; SD: standard deviation.

A. Linear Mixed Effects model random effects

	Variance	SD
Site (intercept)	95.66	9.78

B. Type II Wald F tests with Kenward-Roger df

	F	DF	Df.res	p value
AHS	0.42	1	3.59	0.558
Size (diameter in cm)	6.32	2	13.72	**0.011**
Depth (m)	11.24	1	6.50	**0.014**
AHS*Size	0.65	2	13.72	0.535

C**.** Pairwise differences of predicted severe bleaching means in *Acropora* corals for different colony sizes across depth and the mean AHS value, 5.0147.

Pairwise differences of Size (cm)	Estimate	SE	DF	p value
(5-9) - (10-29)	-12.63	4.39	13.7	**0.031**
(5-9) - (≥ 30)	-14.29	4.39	13.7	**0.015**
(10-29) - (≥ 30)	-1.66	4.39	13.7	0.925

D. Pairwise differences of predicted severe bleaching means in *Acropora* corals for different depths across size classes and the mean AHS value, 5.0147.

Pairwise differences of Depth	Estimate	SE	DF	p value
10 m depth – 17 m depth	27.2	8.12	6.5	**0.014**

Supplementary Table S2: Severe bleaching linear mixed model (LMM) results for *Pocillopora* corals. **A)** random effects**, B)** Wald Chi-square test of LMM, and **C)** Pairwise differences in predicted severe bleaching means for *Pocillopora* corals of different colony sizes interacting with accumulated heat stress (AHS). Pairwise comparisons were calculated using a Tukey HSD (honest significant difference) test. SE: standard error; DF: degrees of freedom; SD: standard deviation.

A. Linear Mixed Effects model random effects

	Variance	SD
Site (intercept)	8.33	2.89

B. Type II Wald F tests with Kenward-Roger df

	F	DF	Df.res	p value
AHS	9.60	1	1.84	0.099
Size (diameter in cm)	7.88	2	14.19	**0.005**
Depth (m)	1.61	1	8.33	0.238
AHS*Size	8.60	2	14.19	**0.004**

C. Pairwise differences of predicted severe bleaching means in *Pocillopora* corals for different colony sizes and mean maximum AHS (5.0147) across depth.

Pairwise differences of Size (cm)	Estimate	SE	DF	p value
(5-9; AHS:5.0147) - (10-29; AHS:5.0147)	0.47	5.58	14.2	0.996
(5-9; AHS:5.0147) - (≥ 30; AHS:5.0147)	-18.95	5.58	14.2	**0.011**
(10-29; AHS:5.0147)- (≥ 30; AHS:5.0147)	-19.42	5.58	14.2	**0.010**

References

1. R Core Team (2020). R: A language and environment for statistical computing. R Foundation for Statistical Computing, Vienna, Austria.URL https://www.R-project.org/.
2. Wickham, H. *Ggplot2: Elegant Graphics for Data Analysis*. (Springer, 2016).
3. Hijmans, R. J. raster: Geographic Data Analysis and Modeling. R package version 3.3-13. https://CRAN.R-project.org/package=raster (2020).
